# Disability Trends Among Community-Dwelling Older Adults and Related Determinants of Disability

**DOI:** 10.1093/geroni/igab046.451

**Published:** 2021-12-17

**Authors:** Qiwei Li, Sarah Szanton

**Affiliations:** 1 Johns Hopkins School of Nursing, Baltimore, Maryland, United States; 2 Johns Hopkins University, Baltimore, Maryland, United States

## Abstract

The growing aging population with disabilities poses challenges to caregiving and health care services but there is little recent data on disability trends. Some studies have shown that disability is decreasing while others have shown it increasing. Understanding these trends among community-dwelling older adults is critical for communities to allocate resources and develop policies. This study updates disability trend data among community-dwelling older adults using nationally representative National Health & Aging Trends Study data. Results revealed that about 30% of Medicare beneficiaries had at least one limitation of the activity of daily living (ADL) from 2011 to 2019. Age 75-79 (IRR=1.55), 80-84 (IRR= to 4.60), 85-89 (IRR=2.99), 90+ (IRR=4.60), female, (IRR=1.18), not a house owner (IRR=1.51), financial strain (IRR=1.71), and receiving Medicaid (IRR=1.84) are associated with a higher likelihood of becoming ADL limited or having more ADL limitations. We will discuss potential policy, intervention, and research implications.

